# Editorial Expression of Concern to: Blockade of BFA-mediated apoptosis in macrophages by the HIV-1 Nef protein

**DOI:** 10.1038/s41419-025-07600-5

**Published:** 2025-04-07

**Authors:** W. Abbas, K. A. Khan, A. Kumar, M. K. Tripathy, I. Dichamp, M. Keita, U. Mahlknecht, O. Rohr, G. Herbein

**Affiliations:** 1 Department of Virology, Pathogens & Inflammation Laboratory, University of Franche-Comte, EA 4266, SFR FED 4234, CHRU Besancon, Besançon, F-25030 France; 2 University of Heidelberg Medical Center, St. Lukas Klinik Solingen, Solingen, D-42697 Germany; 3 Institut de Parasitologie et Pathologie Tropicale, EA 4438, Strasbourg University, 3 rue Koeberlé, Strasbourg, 67000 France

Editorial Expression of Concern to: *Cell Death and Disease* 10.1038/cddis.2014.16, published online 20 February 2014

The Editors are issuing this Editorial Expression of Concern to alert readers that Figure 5g appears to partially overlap with Figure 3 in a previously published article by overlapping authors [1].

The corresponding author has stated that because the experiments were conducted more than ten years ago, the original data is no longer available. Readers are urged to take caution when interpreting the content and conclusions of this article. None of the authors has responded to correspondence from the Publisher about this statement.
